# Optimization of Cancer Treatment in the Frequency Domain

**DOI:** 10.1208/s12248-019-0372-4

**Published:** 2019-09-11

**Authors:** Pascal Schulthess, Vivi Rottschäfer, James W. T. Yates, Piet H. van der Graaf

**Affiliations:** 1 LYO-X GmbH, Basel, Switzerland; 2 Systems Biomedicine & Pharmacology, Leiden Academic Centre for Drug Research, Leiden University, 2333 CC Leiden, The Netherlands; 3 Certara QSP, Canterbury Innovation Centre, Canterbury, UK; 4 Mathematical Institute, Leiden University, Leiden, The Netherlands; 5 DMPK, Oncology R&D, AstraZeneca, Chesterford Research Park, Cambridge, UK

**Keywords:** cancer, dosing frequency optimization, frequency-domain response analysis, quantitative systems pharmacology

## Abstract

Thorough exploration of alternative dosing frequencies is often not performed in conventional pharmacometrics approaches. Quantitative systems pharmacology (QSP) can provide novel insights into optimal dosing regimen and drug behaviors which could add a new dimension to the design of novel treatments. However, methods for such an approach are currently lacking. Recently, we illustrated the utility of frequency-domain response analysis (FdRA), an analytical method used in control engineering, using several generic pharmacokinetic-pharmacodynamic case studies. While FdRA is not applicable to models harboring ever increasing variables such as those describing tumor growth, studying such models in the frequency domain provides valuable insight into optimal dosing frequencies. Through the analysis of three distinct tumor growth models (cell cycle-specific, metronomic, and acquired resistance), we demonstrate the application of a simulation-based analysis in the frequency domain to optimize cancer treatments. We study the response of tumor growth to dosing frequencies while simultaneously examining treatment safety, and found for all three models that above a certain dosing frequency, tumor size is insensitive to an increase in dosing frequency, e.g., for the cell cycle-specific model, one dose per 3 days, and an hourly dose yield the same reduction of tumor size to 3% of the initial size after 1 year of treatment. Additionally, we explore the effect of drug elimination rate changes on the tumor growth response. In summary, we show that the frequency-domain view of three models of tumor growth dynamics can help in optimizing drug dosing regimen to improve treatment success.

**Electronic supplementary material:**

The online version of this article (10.1208/s12248-019-0372-4) contains supplementary material, which is available to authorized users.

## INTRODUCTION

In clinical pharmacology, treatment regimen are usually defined by drug dose, dosing interval, and treatment duration. Because the success of drug interventions heavily depends on drug administration schedules, the high rate of late-stage attrition in clinical development can be attributed partly to sub-optimal dosing regimen selection [1, 2]. Often, dose and dosing schedule are determined through pharmacokinetic and pharmacodynamic (PKPD) model simulations [3]. In quantitative systems pharmacology (QSP), such PKPD models are combined with mechanistic systems biology and/or disease models [4]. Such mechanistic models have long been used to describe and predict various aspects in oncology [5–7], from the underlying biological mechanisms [8–10] to tumor growth [11, 12]. While QSP is increasingly utilized in anti-cancer drug discovery and development [13, 14], only a few examples exist where it has been applied to optimize drug dosing and scheduling to predict tumor responses, efficacy, and toxicity [15, 16]. Control theory methods, but almost exclusively optimal control theory, have been used to optimize dosing regimen [17, 18]; however, the range of analyzed regimen has been limited. Primarily, optimal control theory served as a method to optimize dose [19–21]. Fister and Panetta [22], for example, used optimal control theory on a model of cell cycle-specific bone marrow growth to determine effective administrations of a chemotherapeutic agent while maximizing bone marrow mass and the drug dose over the treatment interval.

However, there has been little systematic effort in determining the influence of dosing frequency on treatment success by using PKPD models. To approach this gap, we recently published a tutorial for pharmacologists on frequency-domain response analysis (FdRA), an analytical method commonly used in systems and control engineering [23]. QSP models relate inputs such as the plasma concentration of a drug or a schedule of drug administrations to outputs such as the effect of a drug. Because these key variables typically vary in time, QSP models are often based on differential equations. The time scales on which they act can differ significantly, from drug-receptor binding happening within seconds to tumor growth over the course of years. Similarly, disturbances of dynamic biological systems, such as drug interventions, can span multiple time scales as well. FdRA provides a framework for analyzing how such disturbances on various time scales affect dynamic systems by focusing on the change of the harmonic content (i.e., frequency, amplitude, and phase) of an input signal when it is passed to the output, rather than its temporal evolution. Additionally, in combination with (preclinical) high-throughput dose-exposure-response experimentation, FdRA allows for the identification of a system’s structure describing, e.g., the dynamic connection between dose and response without requiring prior biological or pharmacological knowledge [24, 25]. FdRA, however, requires a model to be linear or at least linearizable around a stable steady state. Consequently, FdRA is not applicable to models that do not possess a stable steady state because they, for example, contain monotonically increasing variables as present in tumor growth models. Nevertheless, we demonstrated earlier that non-linear models and their linearization’s lead to comparable frequency responses [23].

Here, we present a simulation study that is heavily inspired by FdRA in that it shifts the focus away from the traditionally used time domain towards the frequency domain with the aim to find the frequency response behavior of three models of tumor growth to chemotherapeutic treatment, and so to suggest optimal dosing regimen.

**Fig. 1:**
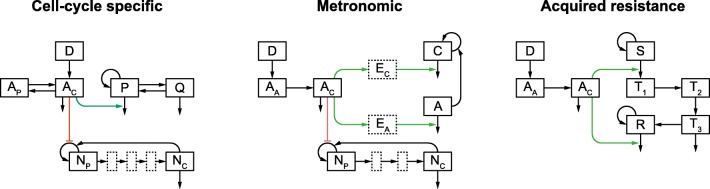
Model structures. For the three models (CCSM, MM, and ARM), the model structures are shown. Green and red arrows denote stimulation and inhibition, respectively. Model variables are abbreviated as the following: D is the drug dose; A_A_, A_C_, A_P_ are the drug concentrations in the absorption, central, and peripheral compartments, respectively; P and Q represent proliferating and quiescent cancer cells; E_C_ and E_A_ are the effect compartment for cancer cells and the anti-angiogenic effect, respectively; C and A are the cancer cells and the anti-angiogenic effect, respectively; N_P_ and N_C_ represent proliferating and circulating neutrophils, respectively; S represents sensitive cells; T_1_ to T_3_ are cells in different stages of damage; R is the resistant cells

The three selected models capture essential aspects of chemotherapy, namely the cell cycle specificity and the anti-angiogenic effects as well as the development of resistance to chemotherapeutic agents (Fig. 1). Cell cycle-specific chemotherapy has been well-studied with the help of mathematical models [26]. Dibrov *et al.*, for example, studied the frequency dependence of cell cycle-dependent chemotherapy with the help of (optimal) control theory methods already more than 40 years ago [27, 28]. While they included a drug concentration dependence in their model, pharmacokinetics (PK) is not fully incorporated. Similarly, Agur *et al.* [29] modeled cell cycle kinetics in normal and tumor tissue to optimize pulsatile dosing without the inclusion of PK. And lastly, also Bernard *et al.* [30] studied the impact of variations of tumor cell kinetics on anti-cancer chronotherapy without the addition of a PK model. Here, we use a cell cycle-specific model (CCSM) that divides human tumor cells into proliferating cells in G1, S, G2, or M phase and quiescent cells in G0 phase of the cell cycle [31]. It is, furthermore, coupled to a two-compartmental PK model of etoposide and a myelosuppression model to predict toxicities [32]. Metronomic chemotherapy, and anti-angiogenesis as its main mode of action, is well studied from a theoretical perspective as well [33–36]. With the help of a mathematical model, Mpekris *et al.*, for example, found that metronomic chemotherapy improves the vascular perfusion of tumor tissue which resulted in improved drug delivery and higher tumor cell kill rates [37]. The metronomic model (MM) used in this article combines a PK model of temozolomide with a tumor growth model that contains a description of the anti‐angiogenic effect, and a model of myelosuppression [38]. The prevention of resistance to anti-cancer therapies with the optimization of dosing schedules has been excessively examined using mathematical models [39–41]. Here, we analyze a murine PKPD model of acquired resistance (ARM) of tumor cells in response to erlotinib or gefitinib treatment [42].

We study the response of each of these three tumor growth models to different dosing frequencies, the interplay of PK and dosing regimen on treatment success, and the optimal treatment modality to maximize tumor reduction while limiting toxicities. Not surprisingly, we find that PK significantly impacts the success of treatment; however, the frequency-domain view identifies previously unknown deviations from conventional dosing regimen. Additionally, we suggest modified drug elimination rates for next-generation compounds that would lead to optimal tumor reduction with acceptable toxicity.

Thus, we show that analyzing QSP models in the frequency domain not only provides insights into the dynamics of tumor growth and their response to repetitive treatments, but also allows to detect optimal dosing regimen for given drug behaviors.

## MATERIALS AND METHODS

All models were implemented as described in the respective publications and verified by reproducing the published simulations. Here, it should also be noted that between-subject variability in the PK parameters was not considered.

### Cell Cycle-Specific Model

The cell cycle‐specific model (CCSM) by Zhu *et al.* [18] combines a two‐compartmental PK model of etoposide in humans, a tumor growth model, and a myelosuppression model (Fig. 1 left). Etoposide is administered into the central compartment (*A*_*C*_) and can distribute into a peripheral compartment (*A*_*P*_). The tumor growth model divides tumor cells into two compartments—proliferating cells in G1, S, G2, or M phase (*P*) and quiescent cells in G0 phase of the cell cycle (*Q*). It is assumed that quiescent cells are not affected by etoposide. The myelosuppression model describes the maturation of proliferating stem and progenitor cells in the bone marrow (*N*_*P*_) into circulating neutrophils (*N*_*C*_). Etoposide stimulates the degradation of proliferating tumor cells and inhibits regeneration of stem cells. The model equations and parameters are given in the Supplementary Text. The effect of drug concentration on cell killing (parameter *k*_1_) was not reported by Zhu *et al.* [18], and therefore, set to *k*_1_ = 0.8 d^−1^ based on a cell cycle-specific model of breast cancer data by Panetta and Adam [43].

### Metronomic Model

The metronomic model (MM) describes the effect of temozolomide on tumor growth as well as its anti-angiogenic effect [44] (Fig. 1 middle). A myelosuppression model of temozolomide was first developed by Panetta *et al.* [45], and coupled to the tumor growth model by Houy and Grand [38]. Temozolomide is administered orally as represented by the absorption compartment (*A*_*A*_) after which it distributes into the central compartment (*A*_*C*_). The effect of temozolomide on tumor size (*C*) and angiogenesis (*A*) is mediated by two effect compartments (*E*_*C*_ and *E*_*A*_). The maturation of proliferating stem and progenitor cells in the bone marrow (*N*_*P*_) gives rise to neutrophils in circulation (*N*_*C*_). Temozolomide promotes tumor cell degradation and anti-angiogenesis while inhibiting stem and progenitor cell proliferation. With the originally reported parameters, the main findings of Faivre *et al.* [44] could not be reproduced. We, therefore, digitized their key figures and estimated a new parameter set (Supplementary Figure S1). The model equations and the original as well as the newly estimated parameters are reported in the Supplementary Text.

### Acquired Resistance Model

Eigenmann *et al.* [42] recently developed a murine tumor growth inhibition model that describes the killing of tumor cells in response to erlotinib or gefitinib treatment and the formation of resistant cells (Fig. 1 right). An EGFR inhibitor, erlotinib or gefitinib, is administered orally into an absorption compartment (*A*_*A*_) and distributes into the central compartment (*A*_*C*_). In response to drug treatment, sensitive cells (*S*) undergo several stages of damage (*T*_1_ to *T*_3_) and are either killed or converted to resistant cells (*R*). Eigenmann *et al.*, however, also assume that a threshold of drug plasma concentration exists above which the drugs are affecting the resistant cells. This threshold concentration is derived from an *in vitro* threshold by correcting for fraction unbound in plasma [46, 47]. The model equations, as well as the parameter sets for erlotinib and gefitinib in mouse, are given in the Supplementary Text.

### Frequency-Domain Response Analysis

Recently, FdRA was introduced to a pharmacometrics audience [23]. FdRA analytically determines how the frequency of an input modulates the output behavior of a linear dynamic system. Following a steady-state analysis, a non-linear model, such as those usually present in QSP, is linearized around a stable steady state after which the frequency response can be determined. Because tumor growth models usually grow indefinitely, no stable steady state can be found other than a trivial steady state at the origin. Thus, a linearization is not possible, and FdRA cannot be employed. Therefore, we performed a simulation study that mimics FdRA numerically by simulating the time courses of tumor growth, absolute neutrophil count (ANC), and the amplitude ratios between tumor and PK for a large range of dosing frequencies. Afterwards, we plotted tumor growth, ANC, and amplitude ratios after a certain treatment duration over the frequency of dose administration. Here, it should be noted that FdRA only allows for symmetric dosing regimen (e.g., daily, weekly) which means that irregular dosing regimen such as administration on five consecutive days once per month or different dose amounts such as loading doses cannot be visualized in the frequency response graphs. Additionally, we performed an analytical analysis of the metronomic model in order to obtain explicit and approximate solutions of the differential equations with which we could substantiate the numerical findings (Supplementary Text).

### Doses

Throughout this article, we maintain constant total drug exposure to avoid that more frequent dosing results in a greater drug exposure, and thus to make results across different dosing regimen comparable. Conventionally, etoposide and temozolomide are administered on five consecutive days once per month at single doses of 100 mg m^−2^ and 200 mg m^−2^, respectively. Thus, a 70-kg patient with a body surface area of 1.8 m^2^ will receive 10.8 g etoposide or 21.6 g temozolomide per year. Erlotinib and gefitinib are administered daily at doses of 100 mg kg^−1^ and 150 mg kg^−1^, respectively. Mice with a body weight of 25 g, thus, receive 7.58 mg erlotinib or 11.42 mg gefitinib per month. The dose administered at each treatment is adjusted to keep the total dose, and so total exposure to a drug, constant. A daily administration of etoposide would, for example, calculate to a single dose of 29.6 mg whereas a dose of 207.7 mg would be administered in a weekly schedule.

### Safety Determination

The cell cycle-specific and the metronomic model both contain a myelosuppression model that allows the determination of treatment safety in terms of ANC. No such model exists relating adverse events to epidermal growth factor receptor (EGFR) inhibition. We normalize ANC levels to ANC before treatment and determine two safety measures. By measuring minimum ANC, we assume that ANC levels below 6% represent neutropenia [33]. Furthermore, we determine the ability of neutrophil levels to recover between two doses by measuring maximum ANC levels just before the administration of the next dose.

### Software

All simulation results were obtained with R 3.5.0. For the parameter estimation of the metronomic model, MATLAB R2018a was used. Mathematica 11.3.0 was consulted for the analytical analysis of the metronomic model.

## RESULTS

### Tumor Development

**Fig. 2:**
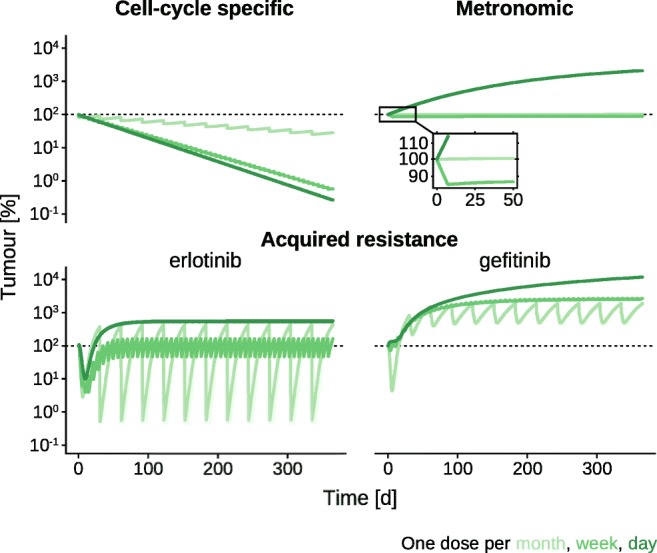
Tumor development over time at different dosing frequencies. For the three models (CCSM, MM, and ARM), tumor cell count (CCSM), tumor mass (MM), and tumor volume (ARM) with respect to initial tumor cell count/mass/volume before treatment in logarithmic percent are plotted over 1 year of repetitive dose administration every month (light green), week (medium green), and day (dark green)

For all three models (CCSM, MM, and ARM), the time-resolved change in tumor tissue in response to three repetitive dosing schemes (one dose every month, week, and day) over the course of 1 year is shown in Fig. 2. All three dosing schemes applied to the cell cycle-specific model lead to a reduction of the number of tumor cells after 1 year (Fig. 2 top left). Daily and weekly doses of etoposide result in a tumor cell count reduction of 99.7% and 99.4% after 1 year, respectively, while a monthly dose reduces the number of tumor cells by 71.5% as compared to tumor cell mass prior to treatment. Additionally, ANC fluctuations change in response to dosing frequency (Supplementary Figure S2). A monthly schedule brings ANC close to levels observed in neutropenia which, however, recover again to 39.6% above pre-treatment ANC levels before the next dose is administered. Daily and weekly administrations result in a sustained reduction of ANC levels to 42.2% of the initial ANC level with only slight or no fluctuations.

For the metronomic model, a daily administration schedule results in a large increase in tumor mass while the monthly dosing schedule increases tumor mass by 1.2% after 1 year (Fig. 2 top right). Only the administration of one dose of per week leads to a slight decrease in tumor mass with a maximum decrease by 14.3% after 7 days of treatment, and a decreased by 11% after 1 year. An analytical analysis of this model, furthermore, confirms these findings (Supplementary Text and Supplementary Figure S3). There, we either solve the differential equations analytically or approximate their solutions which results in a very good correspondence. For a daily dosing regimen, we analytically find that tumor mass increases indefinitely, while it stays nearly constant in a monthly regimen. Administering temozolomide every 5 days displays a significant decrease in tumor mass. Lastly, an increased dosing frequency also reduces the fluctuations and mean ANC levels with a daily dose administration leading to ANC levels of less than 6% after 22 days whereas the monthly administration scheme almost allows for full recovery to pre-treatment ANC levels (Supplementary Figure S2). For the originally reported parameters, the metronomic model does lead to more pronounced reduction of tumor mass for low-frequency administration schedules (Supplementary Figure S4).

Shortly after the start of erlotinib treatment to the acquired resistance model, tumor volume decreases for all dosing frequencies (Fig. 2 bottom left). Low-frequency dosing, however, develops sustained oscillations around the pre-treatment tumor volume with larger amplitudes observed for monthly as compared to weekly treatments. Thus, while an erlotinib dose initially leads to tumor reduction, tumor volume increases prior to the next dose. Only daily administration of erlotinib results in a sustained tumor volume increase after 1 year. Gefitinib administration results in tumor volume increase irrespective of dosing frequency with the largest increase observed for daily interventions (Fig. 2 bottom right). Weekly administrations fluctuate between 2407.4 and 2714.5%. The monthly administration schedule results in tumor volume fluctuations between 489.8 and 1873.0%. We, furthermore, observe that already after 1 month of erlotinib treatment, all sensitive cells have become resistant **(**Supplementary Figure S5). For gefitinib treatment, however, this conversion happens more gradually. A monthly dosing regimen displays large fluctuations in the fraction of resistant cells.

### Tumor Growth Response to Dosing Frequency Changes

**Fig. 3:**
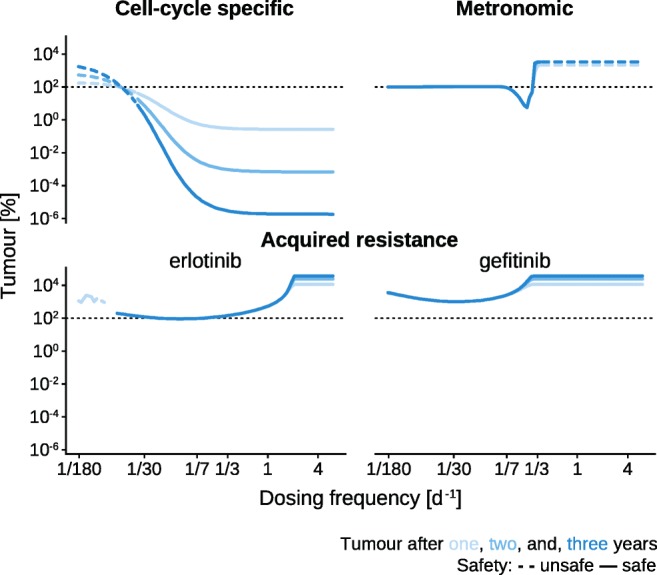
Tumor response to dosing frequency changes. For the three models (CCSM, MM, and ARM), tumor cell count (CCSM), tumor mass (MM), and tumor volume (ARM) with respect to initial tumor cell count/mass/volume before treatment in logarithmic percent are plotted over the frequency of dose administrations after one (light blue), two (medium blue), and three (dark blue) years of treatment. Safe treatments (ANC > 6%) are represented by solid lines, unsafe treatments by dashed lines

To assess the impact of dosing frequency, we calculate the tumor properties for all three models (CCSM, MM, and ARM) after 1, 2, and 3 years of repetitive drug treatment at increasing dosing frequencies (Fig. 3). Each model is excited with drug doses at increasing frequencies after which the mean amount of tumor tissue of the last two dose administrations is divided by the amount of tumor tissue prior to treatment and expressed in percent. For CCSM and MM, the safety of each dosing regimen is, furthermore, determined by calculating ANC as a percental relation between the neutrophil count after 1, 2, and 3 years to the neutrophil count at the beginning of treatment. A 6% ANC nadir is used as a lower threshold for safety [33]. It has been reported that a rash was the most common adverse event when patients with non-small cell lung cancer were treated with 150 mg erlotinib [48] or with 250 mg gefitinib per day [49]. Hence, it is assumed that our use of constant exposure dosing regimen for the acquired resistance model is safe for the majority of animals as well. Stimulating the cell cycle-specific model with prolonged high-frequency etoposide administration results in a reduction of tumor tissue by 99.7% after 1 year, and complete eradication after 3 years (Fig. 3 top left). Infrequent treatment at, for example, one dose every 3 months on the other hand initially leads to a tumor increase by 44.6% after 1 year, and an increase of 524.1% after 3 years. Interestingly, the reduction of tumor cell count did not change between one dose every 3 days and six doses per day. Administering less than one dose every 36 days results in an ANC below 6% and thus, unsafe treatment whereas more frequent dosing is predicted to be a safe treatment regimen (Supplementary Figure S6). On the other hand, when looking at neutrophils’ ability to recover as safety determinant by measuring peak ANC levels in response to dosing frequencies, we observe that dosing more frequently than once every 10 days dropped maximum pre-dose ANC levels below 50% of their levels before treatment.

Administering temozolomide to the metronomic model less frequently than one dose per week leads to a slight increase of tumor mass between 0.2 and 3.3% (Fig. 3 top right). More than two doses per week largely increase tumor mass already after 1 year. This is also the only regimen where a difference of tumor mass over time compared to no treatment can be observed. A dosing regimen between one and three doses per week leads to tumor reduction with the maximum tumor reduction of 94.6% after 1 year observed at one dose every 4 days. A dosing frequency above one dose per 3 days introduces ANC trough levels of 6% or less. The ANC peak describing the ability of neutrophils to recover is at 101% for low-frequency dosing schemes such as one dose every half a year, drops below 50% for one dose every 22 days, and reaches neutropenia levels (< 6%) when the dosing frequency is higher than one dose every 3 days (Supplementary Figure S6). With the exact and approximated analytical solutions of the model, we find that for dosing frequencies higher than 0.32 doses per day, the tumor mass approaches its tumor size limit of 1 kg. For lower dosing frequencies (less than 0.14 doses per day), it can be shown that the tumor mass hardly deviates from its pre-treatment value (Supplementary Text).

The model of acquired resistance mainly responds with an increase in tumor volume irrespective of dosing frequency or drug (Fig. 3 bottom). Only erlotinib dosing at frequencies between one dose every 7 and one dose every 17 days results in a maximal tumor reduction by 7.8% after 3 years. Increasing the dosing frequency above two erlotinib doses per day or one gefitinib dose every 4 days plateaus the tumor volume, i.e., administering gefitinib once every 3 days and six times per day leads to a similar tumor response. Furthermore, only in these plateaued dosing frequency regimes a slight difference in tumor volume after 1 and 3 years is observed. Administering gefitinib less frequently than once every 4 days decreases the tumor volume with a minimum at one dose every 27 days when measured after 3 years after which tumor volume increases again.

### Tumor Growth Response to Dosing Frequency and Elimination Rate Changes

**Fig. 4:**
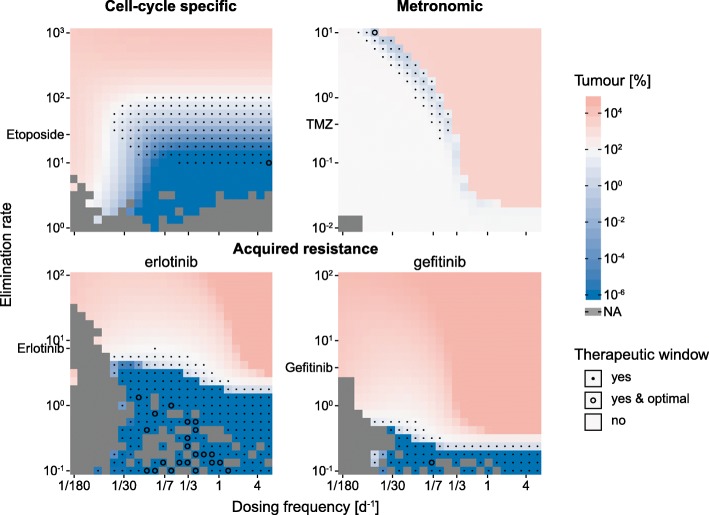
Tumor response to dosing frequency and elimination rate changes. For the three models (CCSM, MM, and ARM), tumor cell count (CCSM), tumor mass (MM), and tumor volume (ARM) with respect to initial tumor cell count/mass/volume before treatment in percent are plotted over the frequency of dose administrations and elimination rates. The tumor response is depicted along a blue to white to red color gradient. Not applicable simulations are given in gray. The therapeutic window is highlighted with black dots while the optimal combination of dosing frequency and elimination rate is denoted as a hollow circle. The elimination rates are given in L day^−1^ (CCSM), h^−1^ (MM), and day^−1^ (ARM)

The interplay between dosing frequency and elimination rate, and its effect on tumor growth is analyzed in Fig. 4. For all three models (CCSM, MM, and ARM), the mean change in tumor tissue after 3 years from its baseline value is calculated for a range of dosing frequencies and two orders of elimination rate magnitudes around the elimination rates of the used drugs. We, furthermore, superimposed the therapeutic window, i.e., dosing frequency and elimination rate combinations that lead to treatments that not only reduce tumor tissue but are also predicted to be safe. For CCSM and MM, treatments are assumed to be safe if ANC levels stayed above 6% while treatments for ARM are always predicted to be safe. The dosing frequency and elimination rate combinations that result in the most pronounced reduction of tumor tissue are highlighted with hollow circles.

In the case of the cell cycle-specific model, lower elimination rates and higher dosing frequencies are more beneficial to tumor reduction (Fig. 4 top left). The therapeutic window is scattered around the original elimination rate of etoposide for mid- to high-frequency dose administrations. The optimal treatment regimen that leads to the largest tumor reduction within the therapeutic window is found at 6 doses per day for an etoposide-like drug with an elimination rate of 10 L day^−1^ (rather than 27.36 L day^−1^).

The metronomic model continues to display the very narrow dosing frequency band that leads to tumor reduction for different elimination rates whereas higher dosing frequencies and elimination rates result in strong increases in tumor response (Fig. 4 top right). Low-frequency dosing and small elimination rates fall short of altering tumor mass. The optimal treatment modalities are found for bi-monthly dose administration with a temozolomide-like drug with an elimination rate of 10 h^−1^ (rather than 0.39 h^−1^). Toxicities are also less pronounced compared to the conventional treatment modalities (Supplementary Figure S7).

Only a reduction of the elimination rate results in tumor reduction in the acquired resistance model (Fig. 4 bottom). As a result, the therapeutic window is also confined to smaller elimination rates. Multiple combinations of smaller elimination rates and dosing frequencies lead to tumor eradication by an erlotinib-like compound. Administering a gefitinib-like compound with an elimination rate of 0.13 day^−1^ (rather than 3.87 day^−1^) once per week minimizes tumor volume.

When comparing tumor development over the course of 4 months of the conventional treatment regimen with the above-identified optimal treatment modalities, we reassuringly observe that the optimal combinations of dosing frequency and elimination rate lead to faster tumor reduction or even eradication (Supplementary Figure S8).

### Amplitude Response to Dosing Frequency Changes

**Fig. 5:**
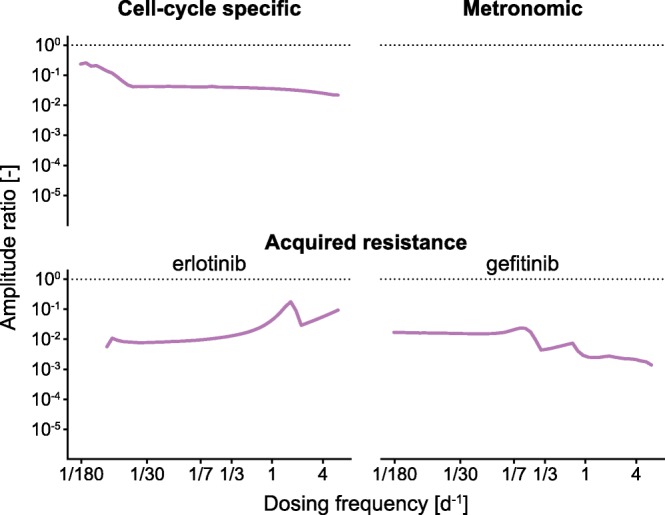
Amplitude response of the tumor to dosing frequency. For the three models (CCSM, MM, and ARM), the amplitude ratio between tumor fluctuations and drug plasma concentration fluctuations is plotted over the frequency of dose administrations

In order to assess the relationship between the fluctuations in tumor tissue (output) to fluctuation in drug plasma concentration (input), we calculate the input/output dosing frequency response (Fig. 5). For that, we determine the ratio of the amplitudes of the tumor and the drug plasma concentration during the last year of a 3-year treatment for a range of dosing frequencies. Thus, an amplitude ratio of 0.1 means that the plasma concentration fluctuations are ten times as high as the tumor fluctuations. Irrespective of model, we observe that the amplitude ratios always stayed below 1. In other words, in all models, the drug plasma concentration fluctuations are always attenuated by the model and lead to smaller fluctuations in the tumor.

For the cell cycle-specific model, the input/output response stays within one order of magnitude with a larger amplitude ratio for low-frequency dosing (Fig. 5 top left). When determining the response of ANC fluctuations to plasma fluctuations, we observe a peak in the amplitude ratio when dosing once every 29 days, and a constant amplitude ratio above dosing frequencies of one dose every 3 days (Supplementary Figure S9).

Because the tumor mass in the metronomic model does not fluctuate, the input/output response is zero for all dosing frequencies (Fig. 5 top right). However, the input/output response can be determined when using ANC levels as output which shows a steep drop of the amplitude ratio over 18 orders of magnitude up until a dosing frequency of one dose per day when ANC levels become zero (Supplementary Figure S9).

In the case of the acquired resistance model, we observe an increasing amplitude ratio when administering erlotinib, and a decreasing amplitude ratio when treating with gefitinib for increasing dosing frequencies (Fig. 5 bottom).

## DISCUSSION

By numerically studying three models of tumor growth and treatment effects that capture essential aspects of chemotherapy, we unraveled their response behavior to chemotherapeutic treatment in the frequency domain.

For the cell cycle-specific model, we found that even though more frequent dose administration is favorable to tumor reduction, it also results in a sustained reduction of ANC levels while less frequent etoposide administration allows for a strong ANC recovery. This trade-off between efficacy and safety was also observed by Andersen and Mackey [50] who investigated resonance in periodic chemotherapy for acute myelogenous leukemia, and found that this type of intervention is unlikely to be efficacious because tumor cells seem to be favored over bone marrow cells in terms of depletion and regrowth rate. Through adaptation of PK, we furthermore found an etoposide-like drug with a reduced elimination would rather accelerate tumor reduction while still residing within the therapeutic window. This suggests that a mid-frequency dosing regimen might be optimal when describing tumor growth with a cell cycle-specific model.

The metronomic model, although specifically developed for frequent low-dose drug administrations, is not able to confirm this treatment regimen but on the contrary suggests that infrequent treatments should be preferred in terms of tumor reduction and toxicity. While maximum tumor reduction might be achieved for four temozolomide administrations per week, ANC levels below 10% are not a tolerable adverse effect. However, a temozolomide-like therapeutic agent with an increased elimination rate that is administered only every 2 months results in fast tumor reduction with only mild toxicities. In line with the here presented conclusions, two studies of anti-angiogenic chemotherapy, however without PK, investigated the treatment frequency and concluded that the efficacy of metronomic therapy depends the interplay of the vascular contribution to tumor growth and the anti-angiogenic effect of the therapy [51, 52]. This confirms the importance and impact of dosing frequency on the success of metronomic chemotherapy. We, furthermore, showed how explicit or approximate analytical analysis can deepen the insight gained from a model, especially with respect to dosing frequencies.

As expected, in the model of acquired resistance, the resistant cells quickly dominate the sensitive cells, irrespective of drug or species. However, the subtle differences in tumor development and resistant cell behavior can be explained by the different model-inherent drug plasma concentration thresholds above which the drugs also affect resistant cells (Supplementary Table S3). Additionally, drug-specific PK parameters are responsible for the different responses of the model to dosing frequency changes. Therefore, reduction of the elimination rates by two orders of magnitude is the only option to combat resistance and achieve tumor reduction while the dosing frequency seems to have only a minor impact.

By looking at the input/output behavior of all three models through the amplitude ratio between tumor cell count/mass/volume and drug plasma concentration, we found that the amplitude ratios of all models stay below 1, which means that the tumor growth models attenuate plasma fluctuations before they are passed to the tumor response. In other words, the amount of tumor tissue always fluctuates less than the amount of drug in plasma. Furthermore, because all amplitude ratios stay within one order of magnitude, we reason that dosing frequency does not significantly alter the relationship between plasma concentration and tumor fluctuations.

Here, it should also be noted that FdRA only allows for periodic and symmetric dosing regimen where the dose for each treatment as well as the dose administration interval is the same. That this might not always lead to the optimal treatment modalities was recently highlighted by Chmielecki *et al.* [53]. They used an evolutionary model of non-small cell lung cancer to predict that high-dose pulses combined with continuous low-dose tyrosine kinase inhibitors such as gefitinib or erlotinib delay the emergence of resistance. This prediction was, however, later refuted in a phase 1 clinical study that found no improvement of progression free survival or prevention of the emergence of resistance [54]. Nevertheless, non-symmetric dosing regimen were also found to optimize treatment outcome in patients with metastatic breast cancer or non-small-cell lung cancer. Traina *et al.* [55, 56] studied the reduction of consecutive oral capecitabine treatment days from 14 to 7 followed by a 7-day rest period based on simulations of a Norton-Simon growth kinetic model. Similarly, in one of the few studies combining PK with the development of acquired resistance, Foo *et al.* [57] concluded that administration of erlotinib in high-dose pulses with low-dose continuous therapy minimized the development of resistance.

The importance of optimizing dosing regimen also extends into immunotherapy or targeted anti-cancer therapy, such as EGFR inhibition. Sachs *et al.* [58] exemplified that for targeted therapeutics such as monoclonal antibodies or immunotherapies that might not exhibit dose-limiting toxicities, conventional maximum tolerated dose derived first-in-human dosing needs an alternative dosing strategy such as biologically efficacious dose. More specifically, a PKPD coupled tumor uptake model for immunocytokine-based cancer immunotherapy predicted that dose-dense administration schedules improve intratumoral drug uptake [59]. For brain tumors, however, it was found that lapatinib should be administered on a continuous daily schedule [60] while the time intervals between PCV (Procarbazine, CCNU, and Vincristine) chemotherapy cycles should be increased [61].

## CONCLUSION

With the increased interest in dosing regimen optimization, it becomes apparent that each drug and each disease exhibits its own optimal treatment modality, and we believe that FdRA, and the numerical analysis of models in the frequency domain as presented in this article, can provide a helpful tool to approach this challenge. Hence, all three models analyzed in this article exhibit their own response behavior to changes in dosing regimen or drug-specific parameters. Nevertheless, we found that conventional treatment modalities leave considerable room for improvement. Thus, in order to guarantee the best possible treatment outcome, special care should be taken in tailoring the PK profiles to the desired PD response. To that end, extending FdRA by considering between-subject variability would allow dosing frequency optimization even on the population level.

## Electronic Supplementary Material

ESM 1 (PDF 8543 kb)
