# Supplementary material for: Optimization of Cancer Treatment in the Frequency Domain
Source: AAPS J. 2019 Sep 11;21(6):106. doi: 10.1208/s12248-019-0372-4 (PMC6739279; doi:10.1208/s12248-019-0372-4)
Supplement: Supplementary file 1 — (PDF 8543 kb) [file 12248_2019_372_MOESM1_ESM.pdf]

Supplementary data

Optimization of cancer treatment in the frequency domain

P Schulthess, V Rottschäfer, JWT Yates, PH van der Graaf

Contents

S1 Supplementary text **3**
S1.1 Model definitions . . . 3
S1.1.1 Cell-cycle specific model . . . 3
S1.1.2 Metronomic chemotherapy model . . . 4
S1.1.3 Acquired resistance model . . . 5
S1.2 Analysis of the metronomic chemotherapy model . . . 5
S1.2.1 Summary . . . 5
S1.2.2 Solutions and estimates . . . 6
S1.2.3 Low dose, high dosing frequency . . . 12
S1.2.4 Higher dose, lower dosing frequency . . . 13
S1.2.5 The equations for $x_8, \ldots, x_{11}$ . . . 13

S2 Supplementary tables **15**

S3 Supplementary figures **18**

S1 Supplementary text

S1.1 Model definitions

S1.1.1 Cell-cycle specific model

The cell-cycle specific model (CCSM) by Zhu et al. [11] contains the pharmacokinetic model of etoposide, a tumour growth model, and the myelosuppression model first introduced by Friberg et al. [5]:

$$\frac{dx_1}{dt} = k_{12}x_2 - (k_{12} + k_{10})x_1 \qquad x_1(0) = \delta \tag{1a}$$

$$\frac{dx_2}{dt} = k_{12}x_1 - k_{21}x_2 \qquad x_2(0) = 0 \tag{1b}$$

$$\frac{dx_3}{dt} = (\alpha - \mu - \eta)x_3 + \beta x_4 - k_1\frac{x_1}{V}x_3 \qquad x_3(0) = 2 \times 10^{11} \tag{1c}$$

$$\frac{dx_4}{dt} = \mu x_3 - \beta x_4 \qquad x_4(0) = 8 \times 10^{11} \tag{1d}$$

$$\frac{dx_5}{dt} = k_t x_5 \left(1 - m\frac{x_1}{V}\right)\left(\frac{x_9(0)}{x_9}\right)^n - k_t x_5 \qquad x_5(0) = 5 \times 10^9 \tag{1e}$$

$$\frac{dx_6}{dt} = k_t(x_5 - x_6) \qquad x_6(0) = 0 \tag{1f}$$

$$\frac{dx_7}{dt} = k_t(x_6 - x_7) \qquad x_7(0) = 0 \tag{1g}$$

$$\frac{dx_8}{dt} = k_t(x_7 - x_8) \qquad x_8(0) = 0 \tag{1h}$$

$$\frac{dx_9}{dt} = k_t(x_8 - x_9) \qquad x_9(0) = 5 \times 10^9 \tag{1i}$$

Herein, x_1 to x_9 describe the amount of drug in the central compartment, the amount of drug in the peripheral compartment, the number of proliferating cancer cells, the number of quiescent cancer cells, the number of proliferating stem and progenitor cells in the bone marrow, the number of maturing cells in three transit compartments, and the number of circulating neutrophils, respectively. The dose δ and the values of the parameters are given in Table S1. The parameter k_1, which was not reported by Zhu et al. [11], describes the effect of drug concentration on cell killing and is bound to an interval between 0 and 1 [4]. Dua et al. [1] reports a value of $k_1 = 0.8\,\mathrm{d}^{-1}$ based on breast cancer data by Panetta and Adam [8].

S1.1.2 Metronomic chemotherapy model

A mathematical model describing the effect of temozolomide on tumour growth as well its anti-angiogenic effect was first introduced by Faivre et al. [3]. A myelosuppression model of temozolomide was developed by Panetta et al. [9] and coupled to the tumour growth model in Equations 2 as used by Houy and Le Grand [7].

$$\frac{dx_1}{dt} = -k_a x_1 \qquad x_1(0) = \delta \tag{2a}$$

$$\frac{dx_2}{dt} = \frac{k_a}{V} x_1 - k_e x_2 \qquad x_2(0) = 0 \tag{2b}$$

$$\frac{dx_3}{dt} = -a_1 e^{-b_1 x_3} x_3 + (x_2 - c_1)\mathcal{H}(x_2 - c_1) \qquad x_3(0) = 0 \tag{2c}$$

$$\frac{dx_4}{dt} = -a_2 e^{-b_2 x_4} x_4 + (x_2 - c_2)\mathcal{H}(x_2 - c_2) \qquad x_4(0) = 0 \tag{2d}$$

$$\frac{dx_5}{dt} = \lambda \log\left(\frac{K}{x_5}\right) x_5 x_6 - u_1 x_3 x_5 e^{-r x_7} \qquad x_5(0) = 3 \times 10^4 \tag{2e}$$

$$\frac{dx_6}{dt} = R - (R + u_2 x_4) x_6 \qquad x_6(0) = 1 \tag{2f}$$

$$\frac{dx_7}{dt} = (x_2 - c_1)\mathcal{H}(x_2 - c_1) \qquad x_1(0) = 0 \tag{2g}$$

$$\frac{dx_8}{dt} = \mathcal{H}\left(K_D - x_2\right) \frac{r_{\max} K_m + r_{\min} x_{11}}{K_m + x_{11}} x_8 - k_1 x_8 \qquad x_8(0) = \frac{k_2}{k_1} x_9(0) \tag{2h}$$

$$\frac{dx_9}{dt} = k_1 x_8 - k_2 x_9 \qquad x_9(0) = \frac{k_3}{k_2} x_{10}(0) \tag{2i}$$

$$\frac{dx_{10}}{dt} = k_2 x_9 - k_3 x_{10} \qquad x_{10}(0) = \frac{k_{\text{el}}}{k_3} x_{11}(0) \tag{2j}$$

$$\frac{dx_{11}}{dt} = k_3 x_{10} - k_{\text{el}} x_{11} \qquad x_{11}(0) = K_m \frac{r_{\max} - k_1}{k_1 - r_{\min}} \tag{2k}$$

States x_1 to x_7 represent the amount of drug in the absorption compartment, the drug concentration in the central compartment, the effect of temozolomide on cancer and endothelial cells, the mass of the tumour, the anti-angiogenic effect of temozolomide, and the area under the concentration time curve. While x_8 represents the number of proliferating cells in the bone marrow, x_9 to x_{11} denote the number of non-proliferating cells in the bone marrow in early and late maturation stages as well as the number of circulating neutrophils, respectively. The dose is given by δ. Additionally, $\mathcal{H}$ denotes the Heaviside function with

$$\mathcal{H}(x) = \begin{cases} 1 & x \geq 0 \\ 0 & x < 0 \end{cases} \tag{3}$$

With the parameters from Faivre et al. [3], we could not reproduce their figures. Furthermore, only a few parameters in the original article parameters contained the necessary units. We, therefore, digitised Figures 1, 4 and 8 from the original article and estimated a new parameter set (Figure S1). The original and estimated parameters as well as the

parameters of the myelosuppression model used by Houy and Le Grand [7] are given in Table S2.

S1.1.3 Acquired resistance model

Eigenmann et al. [2] recently published the following model for erlotinib and gefitinib that includes acquired resistance.

$$\frac{dx_1}{dt} = -k_a x_1 \qquad x_1(0) = \delta \tag{4a}$$
$$\frac{dx_2}{dt} = k_a x_1 - k_e x_2 \qquad x_2(0) = 0 \tag{4b}$$
$$\frac{dx_3}{dt} = \frac{2\lambda_{0d}\lambda_{1d}x_3}{2\lambda_{0d}x_3 + \lambda_{1d}} - k_2 \frac{x_2}{V} x_3 \qquad x_3(0) = y(0) \tag{4c}$$
$$\frac{dx_4}{dt} = k_2 \frac{x_2}{V} x_3 - k_1 x_4 \qquad x_4(0) = 0 \tag{4d}$$
$$\frac{dx_5}{dt} = k_1(x_4 - x_5) \qquad x_5(0) = 0 \tag{4e}$$
$$\frac{dx_6}{dt} = k_1(x_5 - x_6) - k_{sr}x_6 \qquad x_6(0) = 0 \tag{4f}$$
$$\frac{dx_7}{dt} = \frac{2\lambda_{0r}\lambda_{1r}x_7}{2\lambda_{0r}x_7 + \lambda_{1r}} + k_{sr}x_6 - k_2\left(\frac{x_2}{V} - x_{2t}\right)\mathcal{H}\left(\frac{x_2}{V} - x_{2t}\right)x_7 \qquad x_7(0) = 0 \tag{4g}$$
$$y = x_3 + x_4 + x_5 + x_6 + x_7 \tag{4h}$$

with δ denoting the dose while $\mathcal{H}$ represents the Heaviside function as defined in Equation 3. The states x_1 to x_7 correspond to the amount of drug in the depot compartment, the amount of drug in the central compartment, the volume of dividing cancer cells, the volume of damaged cells in three transit compartments, the volume of resistant cells, while y is the total volume of tumour cells, respectively. The initial tumour volume $y(0)$ is $107\,\text{mm}^3$. A threshold concentration x_{2t} is derived from an *in vitro* threshold by correcting for fraction unbound [6, 10]. Eigenmann et al. [2], furthermore, state that the volume of distribution for gefitinib is dose-dependent. For simplicity, we omitted this dose-dependency. The parameters are given in Table S3.

The fraction of resistant cells is calculated by dividing the volume of resistant cells (x_7) by the total tumour volume y.

S1.2 Analysis of the metronomic chemotherapy model

S1.2.1 Summary

For the metronomic model, we solve the first two equations explicitly and get exact expressions for x_1 and x_2 in Section S1.2.2. In the next sections, we determine exact expressions or approximations for the other solutions for all dosing frequencies. When we compare the results to numerical simulations, we see a very good correspondence (cf.

Figure S3). We find that for low as well as high dosing frequencies we can even find simpler approximations than in general. This leads to several observations:

- When, $d < 3.12$, the dose is small and we find that x_5 (the tumour) increases for all time (see Section S1.2.3). Moreover, $x_5(t) \to K$ as $t \to \infty$. We can even find exact expressions for all x_i, $i = 1, \ldots, x_7$ for $d < 2.85$.
- When the dosing frequency is smaller ($d \geq 7$), and hence, the dose is higher, we find in Section S1.2.4 that the tumour x_5 hardly changes from $x_5(0)$.
- Also, we find that for larger d x_5 does not change anymore after several doses (since $\frac{dx_5}{dt}$ becomes small), and so we conclude that one could stop dosing after that point.
- Moreover, for $d < 0.63$, $x_8, \ldots, x_{11}$ all decay to zero. For larger values of d, $x_8, \ldots, x_{11}$ only decay for a short period of time and have sufficient time to recover back to higher values.

S1.2.2 Solutions and estimates

First, we rescale time t to a new time τ by introducing $t = d\tau$. This implies that for the rescaled system, the dose is given at $\tau = 0, 1, 2, 3, \ldots$. Then, the system of equations becomes

$$
\begin{aligned}
\frac{dx_1}{d\tau} &= -dk_a x_1, \\
\frac{dx_2}{d\tau} &= d\left(\frac{k_a x_1}{V} - k_e x_2\right), \\
\frac{dx_3}{d\tau} &= d((x_2 - c_1)\mathcal{H}(x_2 - c_1) - a_1 x_3 \exp(-b_1 x_3)), \\
\frac{dx_4}{d\tau} &= d((x_2 - c_2)\mathcal{H}(x_2 - c_2) - a_2 x_4 \exp(-b_2 x_4)), \\
\frac{dx_5}{d\tau} &= dx_5\left(\lambda x_6 \log\left(\frac{K}{x_5}\right) - u_1 x_3 \exp(-r x_7)\right), \\
\frac{dx_6}{d\tau} &= d(R - x_6(R + u_2 x_4)), \\
\frac{dx_7}{d\tau} &= d(x_2 - c_1)\mathcal{H}(x_2 - c_1), \\
\frac{dx_8}{d\tau} &= d\left(\frac{\mathcal{H}(K_D - x_2)(K_m r_{max} + r_{min} x_{11})}{K_m + x_{11}} - k_1\right) x_8, \\
\frac{dx_9}{d\tau} &= d(k_1 x_8 - k_2 x_9), \\
\frac{dx_{10}}{d\tau} &= d(k_2 x_9 - k_3 x_{10}), \\
\frac{dx_{11}}{d\tau} &= d(k_3 x_{10} - k_{el} x_{11}),
\end{aligned}
$$

Also, we take the coefficients such that these are per day instead of per hour (by multiplying by 24). We do this without changing notation.

In the next sections, we determine the (approximation of) solutions for every time interval $n \leq \tau < n+1$ in between the different doses and introduce the notation

$$x_{in}(\tau),$$

for $n \leq \tau < n+1$, $i = 1, .., 11$ and $n = 0, 1, 2, \cdots$.

The explicit solutions for x_1 and x_2

The first two equations of the model can be solved explicitly. These explicit solutions are given by

$$x_{1n}(\tau) = u_0 e^{-k_a d\tau} \sum_{i=0}^{n} e^{k_a di},$$

for $n \leq \tau < n+1$, $n = 0, 1, 2, \ldots$ and

$$\begin{aligned} x_{2n}(\tau) &= \frac{u_0 k_a}{(k_e - k_a)V} \left(e^{-k_a d\tau} \sum_{i=0}^{n} e^{k_a di} - e^{-k_e d\tau} \sum_{i=0}^{n} e^{k_e di} \right), \\ &= \frac{u_0 k_a}{(k_e - k_a)V} \left(e^{-k_a d(\tau - n)} \frac{1 - e^{k_a d(n+1)}}{1 - e^{k_a d}} - e^{-k_e d(\tau - n)} \frac{1 - e^{k_e d(n+1)}}{1 - e^{k_e d}} \right), \end{aligned}$$

for $n \leq \tau < n+1$, $n = 0, 1, 2, 3 \ldots$. Note that, x_1 and x_2 both do not differ much per time interval $n \leq \tau < n+1$, and hence, we can approximate x_{1n} by the expression in the first time interval after introducing a shift for τ. Thus, we can approximate $x_{1n}(\tau)$ by $x_{10}(\tau + n)$ and $x_{2n}(\tau)$ by $x_{20}(\tau + n)$. We will use this in the following analysis.

To study the equations for $x_3, \ldots, x_7$, we need to know when $x_{20} > c_i$ for $i = 1, 2$ to determine when the Heaviside functions in the equations for x_3, x_4 and x_7 are non-zero. We find that, depending on the size of the dose, there exists a time interval for which $x_{20} > c_i$. However, there also exists a region of doses for which $x_{20} < c_i$ for all τ and $i = 1, 2$.

To show this, we first determine the maximum of x_2 and denote this by τ_{max}. Using this, we find that $x_2(\tau_{max}) = c_1$ is true for

$$u_0 = c_1 V \left(\frac{k_a}{k_e} \right)^{\frac{k_e}{k_a - k_e}}.$$

This corresponds to a dose every $d = \frac{u_0}{59} \approx 3.12$ days. Moreover, $x_{20}(\tau_{max}) = c_2$ holds for

$$u_0 = c_2 V \left(\frac{k_a}{k_e} \right)^{\frac{k_e}{k_a - k_e}},$$

which corresponds to a doses every $d = \frac{u_0}{59} \approx 2.85$ days.

Summarizing,

- for $d \leq 2.85$, $x_2 < c_2 < c_1$ and
- for $2.85 < d < 3.12$, $c_2 < x_2 < c_1$,

whereas

- for $d > 3.12$ there exists a time interval for which $x_{20} > c_i$ for $i = 1, 2$.

We denote this interval by $\tau^*_{1i}(d) < \tau < \tau^*_{2i}(d)$ for $i = 1, 2$. We find that $\tau^*_{1i}(d)$ both lie close to $\tau = 0$ and that $\tau^*_{2i}(d)$ is also small. So, both the intervals where $x_2 > c_i$, $i = 1, 2$ are small.

In the above three cases for d, the analysis of the solutions is different. We study the first two cases in Sections S1.2.3 and S1.2.4, and now focus on the last case.

For the last case when $d > 3.12$, the Heaviside functions can be approximated since the time intervals for which $x_{20} > c_i$ holds are small. We can replace the contributions of the Heaviside terms in the equations for x_3, x_4 and x_7 by adding a constant to x_3, x_4 and x_7 at every $\tau = n, n \in \mathbb{N}$ when a new dose is given. This yields the leading order contribution to these solutions. The constants are found by integrating $x_{20}(\tau) - c_1$ over the time interval $\tau^*_{11}(d) < \tau < \tau^*_{12}(d)$, for the equations for x_3 and x_7 and similarly by integrating $x_{20}(\tau) - c_2$ over the time interval $\tau^*_{21}(d) < \tau < \tau^*_{22}(d)$, for the equation for x_4. Thus, for a dose u_0, we determine

$$I_1(d) = \int_{\tau^*_{11}(d)}^{\tau^*_{21}(d)} x_{20}(s) - c_1 ds = \left[-c_1 s + \frac{u_0}{V(k_a - k_e)} \left(e^{-k_a ds} - \frac{k_a}{k_e} e^{-k_e ds} \right) \right]_{s=\tau^*_{11}(d)}^{s=\tau^*_{21}(d)}$$

and

$$I_2(d) = \int_{t^*_{12}(d)}^{t^*_{22}(d)} x_{20}(s) - c_2 ds = \left[-c_2 s + \frac{u_0}{V(k_a - k_e)} \left(e^{-k_a ds} - \frac{k_a}{k_e} e^{-k_e ds} \right) \right]_{s=\tau^*_{11}(d)}^{s=\tau^*_{21}(d)}.$$

The $I_i(d)$, $i = 1, 2$, can be determined for various d as

d	3.5	4	5	6	7	8	9
$I_1(d)$	0.75	2.58	7.67	13.93	20.94	28.48	36.42
$I_2(d)$	1.71	3.94	9.59	16.27	23.62	31.45	39.64

Estimates for x_3 and x_4

Using the above, we approximate the equations for $x_{3,4}$ by

$$\frac{dx_3}{d\tau} = -da_1 x_3 \exp(-b_1 x_3), \tag{5a}$$

$$\frac{dx_4}{d\tau} = -da_2 x_4 \exp(-b_2 x_4) \tag{5b}$$

where $I_1(d)$, resp $I_2(d)$, is added to x_3, resp x_4, at every $\tau = 0, 1, 2, 3, \ldots l$. These equations can be solved explicitly by separation of variables as

$$Ei(b_1 x_{3n}(\tau)) = -a_1 d\tau + \tilde{y}_{3n}$$
$$Ei(b_2 x_{4n}(\tau)) = -a_2 d\tau + \tilde{y}_{4n},$$

where Ei is the exponential integral $Ei(x) = \int_x^\infty t^{-1} e^t dt$ and

$$\begin{aligned}
\tilde{x}_{30} &= Ei(b_1 I_1) \\
\tilde{x}_{3n} &= Ei(b_1(x_{3n-1}(n) + I_1)) + a_1 dn \\
\tilde{x}_{40} &= Ei(b_2 I_2) \\
\tilde{x}_{4n} &= Ei(b_2(x_{4n-1}(n) + I_2)) + a_2 dn.
\end{aligned}$$

We can approximate Ei so that this yields a linear expression but these approximations do not correspond very well to the numerical simulations. These solutions are also present in the other equations, and therefore, we will determine an approximation that is easier to handle at that point. From now on we focus on x_3 since x_4 is approximated in a similar way.

The idea is that we replace the x_{3n}-term in the exponential in the equation for x_{3n} by $x_{3n-1}(n) + \frac{1}{2} I_1$. The choice of the $\frac{1}{2}$ is quite arbitrary, and hence we can choose it differently, but it should lie in $[0, 1]$. In this way, we already incorporate into the equation the fact that x_3 increases by I_1 at $\tau = n$. Thus, we approximate Equation 5a for $n \le \tau < n + 1$ by

$$\frac{dx_{3n}}{d\tau} = -da_1 x_{3n} \exp(-b_1(x_{3n-1}(n) + \frac{1}{2} I_1)).$$

We can solve this equation by separation of variables with $x_{30}(0) = 0$ and by also assuming that the solution must be continuous at $\tau = n$.

This yields

$$x_{3n}(\tau) = C_{3n} \exp[D_{3n}\tau], \tag{6}$$

for $n < \tau < n + 1, n \ge 0$, where

$$\begin{aligned}
C_{30} &= I_1 \\
C_{3n} &= (x_{3n-1}(n) + I_1) \exp[a_1 dne^{-b_1(x_{3n-1}(n)+0.5I_1)}] \\
D_{30} &= -a_1 de^{-0.5I_1} \\
D_{3n} &= -a_1 de^{-b_1(x_{3n-1}(n)+0.5I_1)}.
\end{aligned}$$

The approximation for x_4 can be found in a similar way and is given by

$$x_{4n}(\tau) = \tilde{B}_n \exp[A_n \tau],$$

for $n < \tau < n+1, n \geq 0$, where

$$\begin{aligned}
\tilde{B}_0 &= I_2 \\
\tilde{B}_n &= (x_{4n-1}(n) + I_2)\exp[a_2 dn e^{-b_2(x_{4n-1}(n)+0.5I_2)}] \\
A_0 &= -a_2 d e^{-0.5I_2} \\
A_n &= -a_2 d e^{-b_2(x_{4n-1}(n)+0.5I_2)}.
\end{aligned}$$

Estimate for x_7

Solution x_7 can be determined explicitly in terms of exponentials as follows for $n + \tau_{11}^*(d) < \tau < n + \tau_{21}^*(d)$

$$\begin{aligned}
x_{7n}(\tau) &= \left[-c_2 s + \frac{u_0}{V(k_a - k_e)}\left(e^{-k_a ds}\sum_{i=0}^{n} e^{k_a di} - \frac{k_a}{k_e} e^{-k_e ds}\sum_{i=0}^{n} e^{k_e di}\right)\right]_{s=n+\tau_{11}^*(d)}^{s=\tau} \\
&= \left[-c_2 s + \frac{u_0}{V(k_a - k_e)}\left(e^{-k_a ds} - \frac{k_a}{k_e} e^{-k_e ds}\right)\right]_{s=\tau_{11}^*(d)}^{s=\tau-n}.
\end{aligned} \tag{7}$$

and

$$x_7(\tau) = x_{7n}(n + \tau_{21}^*(d)),$$

for $n + \tau_{21}^*(d) < \tau < n + 1 + \tau_{11}^*(d)$, such that x_7 is continuous.

We also determine an approximation since x_7 is present in the x_5-equation. In a similar way as we did for x_3, we can approximate Eq 7, and find that a very good estimate is given by

$$x_{7k}(\tau) = (k+1)I_1,$$

for $k < \tau < k+1, k \geq 1$.

Estimate for x_6

After substituting the estimate of Section S1.2.2 for $x_{4n}(\tau)$ into the equation for x_6, we can solve this equation since it then reduces to a linear equation with an inhomogeneous term. The solution of this equation can be determined explicitly which leads to the

following estimate for x_6

$$
\begin{aligned}
x_{6n}(\tau) &= \tilde{C}_{6n}\exp[-d(R\tau + \frac{u_2 B_n}{A_n}e^{A_n\tau})] \\
&\text{where} \\
\tilde{C}_{6n}(\tau) &= dR\int_n^\tau \exp[d(Rs + \frac{u_2 B_n}{A_n}e^{A_n s})]ds + \hat{C}_{6n} \\
&= -dR\frac{(\frac{-du_2 B_n}{A_n})^{-\frac{A_n}{dR}}}{A_n}\left[\Gamma[\frac{dR}{A_n}, \frac{-du_2 B_n}{A_n}e^{A_n\tau}] - \Gamma[\frac{dR}{A_n}, \frac{-du_2 B_n}{A_n}e^{nA_n}]\right] + \hat{C}_{6n} \\
\hat{C}_{60} &= \exp[d\frac{u_2 B_0}{A_0}]x_6(0) \\
\hat{C}_{6n} &= \exp[d(Rn + \frac{u_2 B_n}{A_n}e^{A_n n})]x_{6n-1}(n),
\end{aligned}
$$

where $\Gamma[a, x]$ is the Incomplete Gamma function. To find the constants $\hat{C}_{6n}$, we again use that the solution must be continuous at $\tau = n$.

Estimate for x_5

Finally, we study the equation for x_5 using the estimates for x_{3n}, x_{7n}, and x_{6n}. Moreover, we assume that x_{5n} does not change much in between doses. Hence, we assume that x_{5n} remains close to $x_{5n-1}(n)$ so that we can replace the $\log(\frac{K}{x_{5n}})$ by $\log(\frac{K}{x_{5n-1}(n)})$. We can even replace $\log(\frac{K}{x_{5n}})$ by $\log(\frac{K}{x_5(0)}) \approx \log(34)$ and find that for most doses this yields a good approximation.

Therefore, we approximate the x_5-equation for $n \leq \tau < n+1$ by

$$
\frac{dx_{5n}}{d\tau} = dx_{5n}\left(\lambda x_{6n}\log\left(L_n\right) - u_1 x_{3n}\exp(-rx_{7n})\right),
$$

with $L_n = \frac{K}{x_{5n-1}(n)}$. This equation can be solved by separation of variables, and hence, we can estimate x_5 as

$$
\begin{aligned}
x_{5n}(\tau) &= \tilde{C}_{5n}\exp\left[\int_n^\tau d\left(\lambda x_{6n}(s)\log\left(L_n\right) - u_1 x_{3n}(s)\exp(-rx_{7n}(s))\right)ds\right], && \text{(8a)} \\
&\text{where} && \text{(8b)} \\
\tilde{C}_{50} &= x_5(0) && \text{(8c)} \\
\tilde{C}_{5n} &= x_{5,n-1}(n). && \text{(8d)}
\end{aligned}
$$

The second integral in Eq. 8a can be determined explicitly, by using Eq. 6 and results from Section S1.2.2 as

$$
\int_n^\tau u_1 x_{3n}(s)\exp[-rx_{7n}(s)]ds = u_1\frac{C_{3n}}{D_{3n}}\left(\exp[D_{3n}\tau] - \exp[D_{3n}n]\right)\exp[-r(n+1)I_1].
$$

We plotted above approximations together with results from simulations for $d = 5$ in Figure S3 and these agree very nicely: the approximations which are plotted in red can

hardly be distinguished from the blue numerical results. Moreover, since x_7 becomes large and $x_6 \to 0$ as τ grows, $\frac{dx_5}{d\tau}$ will become small and hence x_5 will not change after a first time period. This is even true when the dosing is stopped. The reason for this is the following. After dosing is stopped $x_7' = 0$, and so x_7 will not change anymore. Also, $x_3' = 0$ and $x_4' = 0$ will become (very) small since x_3 and x_4 are very large. And, x_6 will keep on decreasing to zero. Using all of this in the equation for x_5, we find that $x_5' \approx 0$ and x_5 will no longer change. Therefore, the tumour will not change or grow even when dosing is stopped.

S1.2.3 Low dose, high dosing frequency

When the dose is so small that $x_2 < c_i$ for $i = 1, 2$ hence when $d \leq 2.85$, then the Heaviside functions in the equations for x_3, x_4 and x_7 remain zero for all time. Then, the equations can be solved explicitly and this yields

$$\begin{aligned} x_3(\tau) &\equiv 0 \\ x_4(\tau) &\equiv 0 \\ x_6(\tau) &\equiv 1 \\ x_7(\tau) &\equiv 0, \end{aligned}$$

and x_5 can be found explicitly as

$$x_5(\tau) = K \exp\left[-\log(\frac{K}{x_5(0)})e^{-\lambda d\tau}\right].$$

Therefore, x_5 (the tumor) will grow for all time where $x_5(\tau) \to K$ as $\tau \to \infty$. In Figure S3, we plot for $d = 1$ these exact expressions in red together with the numerical simulations in blue. Obviously these overlap since the obtained expressions are exact.

Moreover, even when $2.85 < d < 3.12$ and so $c_2 < x_2 < c_1$ we find that

$$x_3(\tau) \equiv 0 \quad x_7(\tau) \equiv 0$$

and hence

$$\frac{dx_{5n}}{d\tau} = dx_{5n}\left(\lambda x_{6n} \log\left(\frac{K}{x_{5n}}\right)\right).$$

Therefore, as long as $x_{5n} < K$, and thus $\log\left(\frac{K}{x_{5n}}\right) > 0$, we find that $\frac{dx_{5n}}{d\tau} > 0$ for all τ. Therefore, x_5 will increase for all τ. Note that $x_5 < K$ is true at $\tau = 0$, and so it holds for all $\tau > 0$. Moreover, $x_5(\tau) \to K$ as $\tau \to \infty$ since $\frac{dx_{5n}}{d\tau} \to 0$ as $x_5(\tau) \to K$.

Summarizing, when a dose is given every d days with $d < 3.12$, the tumour x_5 grows for all time where $x_5(\tau) \to K$ as $\tau \to \infty$.

S1.2.4 Higher dose, lower dosing frequency

When the dose is so high that D_{30} and A_0 in Eq. 6 and Section S1.2.2 are small, the x_3 and x_4 can be approximated by

$$x_{3n}(\tau) = I_1(n+1)$$
$$x_{4n}(\tau) = I_2(n+1)$$

This is true for a dosing frequency of $d \geq 10$ although we find that these approximations even agree very well with the results from the numerical simulations for $d \geq 7$ and larger. Then, we can find x_6 as

$$x_{6k}(\tau) = \frac{R}{R + u_2(k+1)I_2} + \tilde{c}_{6k} \exp\left[-d(R + u_2(k+1)I_2)\tau\right], \tag{9}$$

for $k < \tau < k+1$, where

$$\tilde{c}_{60} = -\frac{R}{R + u_2(k+1)I_2}$$
$$\tilde{c}_{6k} = \left(x_{6k-1}(k) - \frac{R}{R + u_2(k+1)I_2}\right) \exp\left[d(R + u_2(k+1)I_2)k\right]$$

Then, the integrals in expression (8a) can be determined explicitly as

$$\int_k^\tau \lambda x_{6k}(s) \log(L_n)\, ds = \lambda \log(L_n) \left[\left(1 + \frac{u_2(k+1)I_2}{R}\right)(\tau - k)\right.$$
$$\left. + \frac{u_2(k+1)I_2}{Rd(R + u_2(k+1)I_2)k} \left(\exp(-d(R + u_2(k+1)I_2)\tau) - \exp(-d(R + u_2(k+1)I_2)k)\right)\right]$$
$$\int_k^\tau u_1 x_{3k}(s) \exp(-r x_{7k}(s)) ds = u_1(\tau - k)(k+1)I_2 \exp(-r(k+1)I_1).$$

Substituting these expressions into Eq. 8a yields x_5. We plot these expressions in red together with the numerical simulations in blue in Figure S3 for $d = 30$. Again, we see a very good agreement.

The reason that this approximation already works well for $d \geq 7$ is that for this choice, I_1 and I_2 are larger. And, when both I_1 and I_2 become large we find that $\frac{R}{R+u_2(k+1)I_2}$ and the exponential term in Eq. 9 both become small, and therefore, $x_6 \approx 0$ for $\tau > 0$. Moreover, x_7 is also large such that both contributions in the equation for x_5 very small. Therefore, x_5 does not change much as time increases and thus $x_5(\tau) \approx x_5(0)$.

S1.2.5 The equations for $x_8, \ldots, x_{11}$

Next, we analyze the equations for $x_8, \cdots, x_{11}$. First, we determine how the Heaviside function contributes for different values of d. Similarly as in Section S1.2.2, we find that $x_{20}(\tau_{max}) = K_D$ for

$$u_0 = K_D V \left(\frac{k_a}{k_e}\right)^{\frac{k_e}{k_a - k_e}},$$

which corresponds to extremely high ($d \approx 1200$) and not realistic values of d. Hence, for all realistic d there exists a time interval $\tau_1^D < \tau < \tau_2^D$ for which $x_2(\tau) > K_D$ and otherwise $x_2(\tau) < K_D$ holds. It turns out that τ_1^D is (again) very close to zero. Also, for larger values of d the time-interval becomes very small. For smaller values of d, τ_2^D lies close to $\tau = 1$ and there even exist values of d for which $x_2(\tau) > K_D$ for all $\tau > \tau_1^D$. This is true when at $\tau = 1$, $x_{20}(\tau) > K_D$. We determine that $x_{20}(\tau = 1) > K_D$ for $d \approx 0.63$. Hence for $d < 0.63$ we conclude that, since τ_1^D is small, we can approximate this case with assuming that $x_2(\tau) > K_D$ for all τ and therefore $\mathcal{H}(K_D - x_2) = 0$ for all τ.

Summarizing,

- for $d \leq 0.63$, $x_2(\tau) > K_D$ and $\mathcal{H}(K_D - x_2) = 0$ for all τ and
- for $d > 0.63$, there exists a time interval $\tau_1^D < \tau < \tau_2^D$ for which $x_2(\tau) > K_D$ and $\mathcal{H}(K_D - x_2) = 0$. Outside this time-interval $\mathcal{H}(K_D - x_2) = 1$.
- for larger values of d the time interval is small and $\mathcal{H}(K_D - x_2) = 1$ for a larger part of the time.

Next, we study the implications of the value of the Heaviside function in the x_8-equation. When $\mathcal{H}(K_D - x_2) = 0$, the x_8 decays and the equation can solved explicitly as

$$x_8(\tau) = c_8 e^{-k_1 \tau},$$

for some constant c_8. In turn all other equations for x_9, x_{10} and x_{11} can be solved explicitly yielding

$$x_{11}(\tau) = c_1 e^{-k_{el}\tau} + e^{-k_2\tau}(c_2 + c_3\tau) + c_4 e^{-k_1\tau},$$

for some constants c_1, c_2, c_3, c_4. This expression also decays for larger values of τ. So for $d \leq 0.63$, we find that x_{11} decays and $x_{11}(\tau) \to 0$ as τ increases.

When $\mathcal{H}(K_D - x_2) = 1$, we find that there exist two fixed point for the $(x_8, x_9, x_{10}, x_{11})$-system, namely at $(x_8, x_9, x_{10}, x_{11}) = (0, 0, 0, 0)$ and at

$$(x_8, x_9, x_{10}, x_{11}) = \frac{(r_{max} - k_1)K_m k_{el}}{(k_1 - r_{min})k_1} \left(1, \frac{k_1}{k_2}, \frac{k_1}{k_3}, \frac{k_1}{k_{el}}\right) = (x_8(0), x_9(0), x_{10}(0), x_{11}(0)).$$

So, the second fixed point and the initial condition exactly coincide. Therefore, when d is large so that $\mathcal{H}(K_D - x_2) = 1$ for almost all τ, we find that we will remain near the fixed point (initial condition) for all τ. So the maximum of $x_{11}(\tau) = x_{11}(0)$. For smaller d but $d > 0.63$, $x_8, \ldots, x_{11}$ first decay before growing again towards the fixed point. However, the solutions have less time to recover for smaller d than for larger d, thus the maximum of x_{11} will be larger for smaller d than for larger d.

S2 Supplementary tables

Name	Description	Value	Unit
δ	Dose	180	mg
k_{10}	Elimination rate	27.36	$\mathrm{L\,d^{-1}}$
k_{12}	Transfer rate	1.44	$\mathrm{d^{-1}}$
k_{21}	Transfer rate	3.36	$\mathrm{d^{-1}}$
α	Growth rate of proliferating cells	0.5	$\mathrm{d^{-1}}$
β	Rate to become proliferating	0.05	$\mathrm{d^{-1}}$
μ	Rate to become quiescent	0.218	$\mathrm{d^{-1}}$
η	Motility rate	0.477	$\mathrm{d^{-1}}$
k_1	Drug effect slope	0.8	$\mathrm{d^{-1}}$
V	Volume of distribution	6	l
k_t	Transit rate	0.768	$\mathrm{d^{-1}}$
m	Slope	0.126	$\mathrm{\mu M^{-1}}$
n	Feedback strength	0.17	-

Table S1: Parameter values of the cell-cycle specific model.

Name	Description	Original value	Estimated Value	Unit
δ	Dose	360		mg
k_a	Absorption rate	2.4		h^{-1}
k_e	Elimination rate	0.39		h^{-1}
V	Volume of distribution	14		L
a_1	-	0.7	0.031	h^{-1}
b_1	-	0.31	0.64	$L\,mg^{-1}$
c_1	Threshold	3.7	9.24	$mg\,L^{-1}$
a_2	-	0.27	0.014	h^{-1}
b_2	-	0.31	0.4	$L\,mg^{-1}$
c_2	Threshold	0.3	8.45	$mg\,L^{-1}$
λ	Proliferation coefficient	2.3×10^{-4}		-
K	Tumour size limit	1×10^{6}		mg
r	-	0.07	0.25	$L\,mg^{-1}$
u_1	-	0.15	0.0088	$L\,mg^{-1}$
R	-	1	0.031	-
u_2	-	0.6	0.016	$L\,mg^{-1}$
k_1	Transition rate	0.046		h^{-1}
k_2	Transition rate	0.0091		h^{-1}
k_3	Transition rate	0.0091		h^{-1}
k_{el}	Neutrophil elimination rate	0.147		h^{-1}
r_{max}	Maximum growth rate	0.13		h^{-1}
r_{min}	Minimum growth rate	0.0445		h^{-1}
K_m	Half-saturation effect	0.009		-
K_D	Dissociation constant	0.009		-

Table S2: Parameter values of the metronomic model. Where no estimated value is given, the original value was used. Units were derived from the model equations.

Name	Description	Erlotinib	Gefitinib	Unit
δ	Dose	2.5	3.75	mg
k_a	Absorption rate	55	55	d^{-1}
k_e	Elimination rate	7.56	3.87	d^{-1}
V	Volume of distribution	0.127	1.4	L
λ_{0d}	Exponential growth rate	0.217	0.217	d^{-1}
λ_{1d}	Linear growth rate	42.8	42.8	$mm^3\,d^{-1}$
λ_{0r}	Exponential growth rate	0.189	0.189	d^{-1}
λ_{1r}	Linear growth rate	37.193	37.193	$mm^3\,d^{-1}$
k_1	Transit rate	1.52	1.51	d^{-1}
k_2	Drug effect slope	0.0921	0.0921	$L\,mg^{-1}\,d^{-1}$
k_{sr}	Transformation rate	7.4×10^{-3}	7.4×10^{-3}	d^{-1}
x_{2t}	Threshold	7.150	7.448	$mg\,L^{-1}$

Table S3: Parameter values of the acquired resistance model.

S3 Supplementary figures

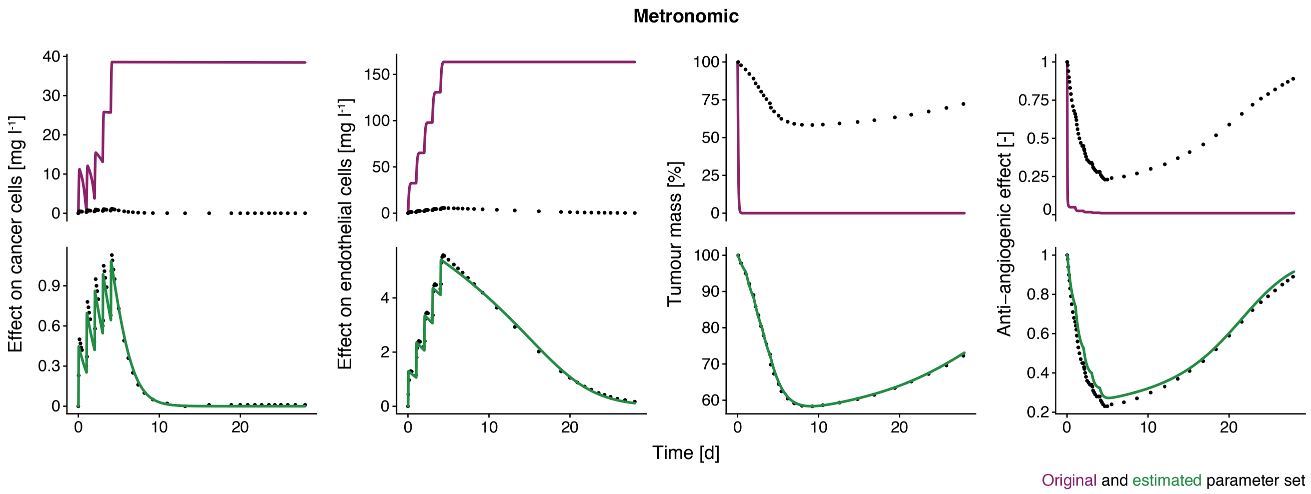

Figure S1: Metronomic model parameter estimation. Black dots represent original digitised data of Figures 1, 4 and 8 in [11] while green lines depict the simulations with the estimated parameter set given in Table S2.

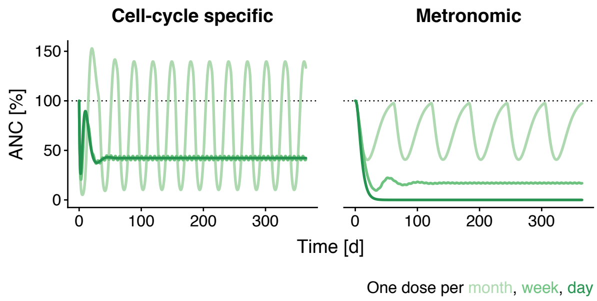

Figure S2: Absolute neutrophil count time course of the cell-cycle specific and the metronomic model for monthly, weekly, and daily administration of etoposide or temozolomide over the course of one year. Drug exposure was kept constant. The dotted lines represent 6 % and 100 % ANC.

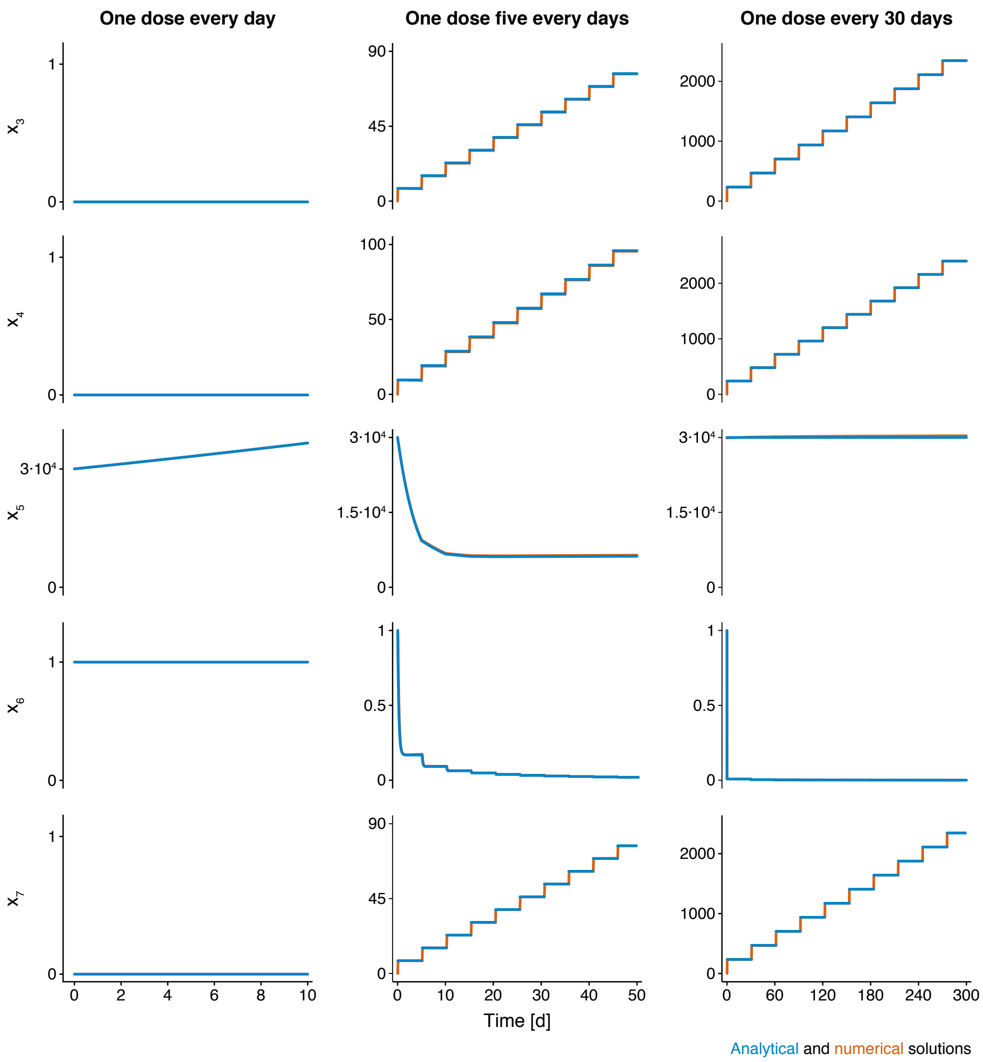

Figure S3: Comparison of (approximated) analytical and numerical solutions of the metronomic model for three dosing regimen. x_3 to x_7 correspond to those defined in Equation 2. We plot explicit expression (where possible) or otherwise the approximations from S1.2.3 (left), S1.2.2 (middle), and S1.2.4 (right).

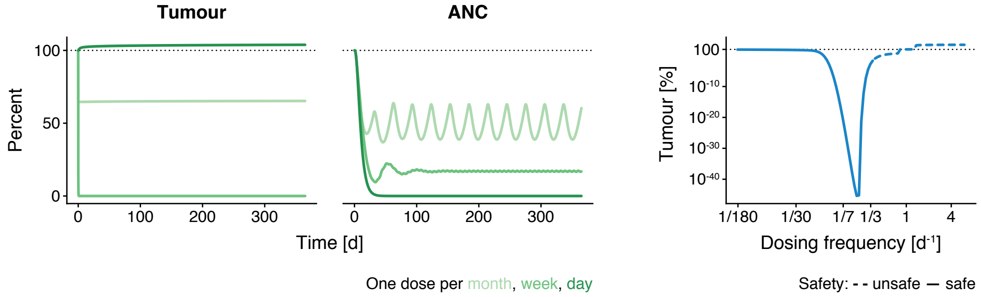

Figure S4: Simulations of the metronomic model with the originally reported parameter set. Left and middle: Time course simulations of the tumour mass and ANC for monthly, weekly, and daily temozolomide administrations over the course of one year. Drug exposure was kept constant. Right: Tumour response to dosing frequency after two years.

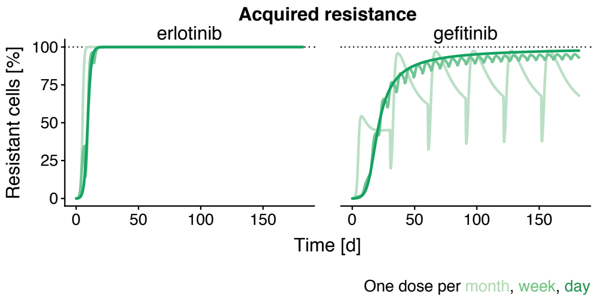

Figure S5: Fraction of resistant cells with respect to total cell volume in the acquired resistance models for monthly, weekly, and daily administration of Erlotinib or Gefitinib to humans or mice over the course of half a year. Drug exposure was kept constant. Dotted lines represent 0 %, 50 %, and 100 % resistant cell fraction.

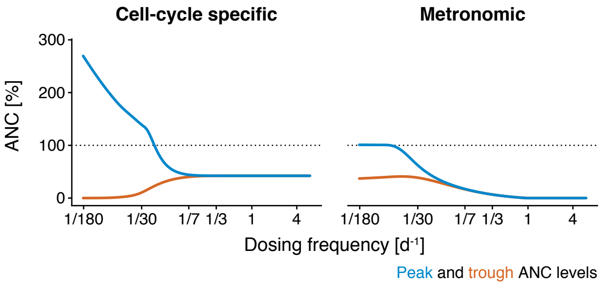

Figure S6: Absolute neutrophil count response of the cell-cycle specific and the metronomic model to dosing frequency after three years. The dotted lines represent 0 % and 100 % ANC.

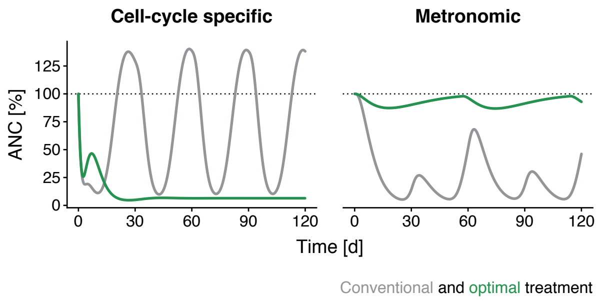

Figure S7: ANC time courses for cell-cycle specific (CCSM) and metronomic models (MM) models comparing conventional and optimal treatment modalities over the course of four month. Conventional treatments are: 180 mg etoposide per day on five consecutive days once per month (CCSM), and 360 mg temozolomide per day on five consecutive days once per month (MM). Optimal treatments are: six 4.9 mg doses of etoposide with an elimination rate of $10\,\mathrm{L\,d^{-1}}$ per day (CCSM), and 3364 mg temozolomide with an elimmination rate of $10\,\mathrm{L\,d^{-1}}$ every two month (MM).

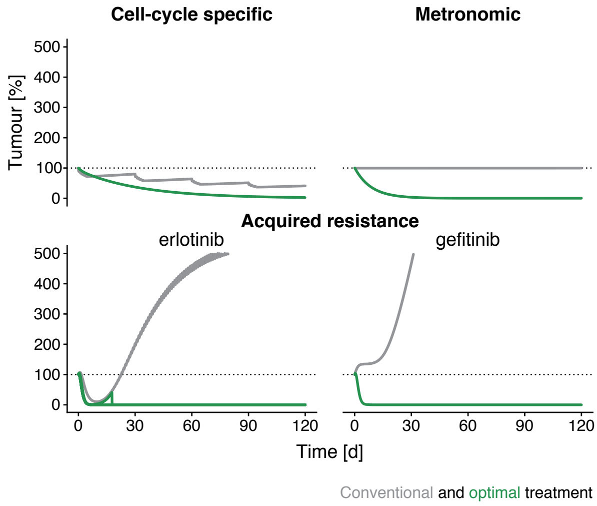

Figure S8: Tumour time courses for cell-cycle specific (CCSM), metronomic (MM), and acquired resistance models (ARM) comparing conventional and optimal treatment modalities over the course of four month. Conventional treatments are: 180 mg etoposide per day on five consecutive days once per month (CCSM), 360 mg temozolomide per day on five consecutive days once per month (MM), 2.5 mg erlotinib per day, and 3.75 mg gefitinib per day. Optimal treatments are: six 4.9 mg doses of etoposide with an elimination rate of $10\,\mathrm{L\,d^{-1}}$ per day (CCSM), 3364 mg temozolomide with an elimmination rate of $10\,\mathrm{L\,d^{-1}}$ every two month (MM), multiple optimal treatments for erlotinib, and 27.7 mg gefitinib every week.

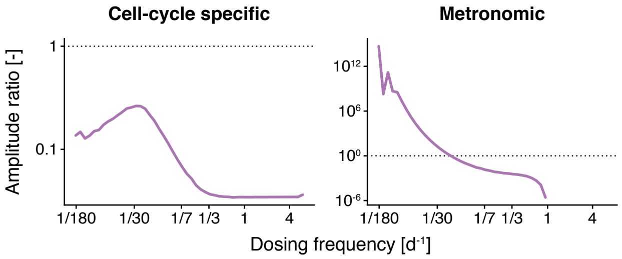

Figure S9: Amplitude response to dosing frequency changes of the cell-cycle specific and the metronomic model with ANC as output. Amplitude ratio is the fraction of ANC amplitude to plasma concentration amplitude. The dotted line represents an amplitude ratio of 1.

Bibliography

[1] P. Dua, V. Dua, and E. N. Pistikopoulos. Optimal delivery of chemotherapeutic agents in cancer. *Computers & Chemical Engineering*, 32(1):99–107, Jan. 2008.

[2] M. J. Eigenmann, N. Frances, T. Lavé, and A.-C. Walz. PKPD modeling of acquired resistance to anti-cancer drug treatment. *Journal of Pharmacokinetics and Pharmacodynamics*, 44(6):617–630, Dec. 2017.

[3] C. Faivre, D. Barbolosi, E. Pasquier, and N. André. A mathematical model for the administration of temozolomide: comparative analysis of conventional and metronomic chemotherapy regimens. *Cancer Chemotherapy and Pharmacology*, 71 (4):1013–1019, Apr. 2013.

[4] K. R. Fister and J. C. Panetta. Optimal control applied to cell-cycle-specific cancer chemotherapy. *SIAM Journal on Applied Mathematics*, 60(3):1059–1072, 2000.

[5] L. E. Friberg, A. Henningsson, H. Maas, L. Nguyen, and M. O. Karlsson. Model of Chemotherapy-Induced Myelosuppression With Parameter Consistency Across Drugs. *Journal of Clinical Oncology*, 20(24):4713–4721, Dec. 2002.

[6] A. Hoshino-Yoshino, M. Kato, K. Nakano, M. Ishigai, T. Kudo, and K. Ito. Bridging from preclinical to clinical studies for tyrosine kinase inhibitors based on pharmacokinetics/pharmacodynamics and toxicokinetics/toxicodynamics. *Drug metabolism and pharmacokinetics*, 26(6):612–620, 2011.

[7] N. Houy and F. Le Grand. Administration of temozolomide: Comparison of conventional and metronomic chemotherapy regimens. *Journal of Theoretical Biology*, 446: 71–78, Mar. 2018.

[8] J. C. Panetta and J. Adam. A mathematical model of cycle-specific chemotherapy. *Mathematical and Computer Modelling*, 22(2):67–82, July 1995.

[9] J. C. Panetta, M. N. Kirstein, A. J. Gajjar, G. Nair, M. Fouladi, and C. F. Stewart. A mechanistic mathematical model of temozolomide myelosuppression in children with high-grade gliomas. *Mathematical Biosciences*, 186(1):29–41, Nov. 2003.

[10] W. Pao, V. A. Miller, K. A. Politi, G. J. Riely, R. Somwar, M. F. Zakowski, M. G. Kris, and H. Varmus. Acquired resistance of lung adenocarcinomas to gefitinib or erlotinib is associated with a second mutation in the EGFR kinase domain. *PLoS medicine*, 2(3):e73, Mar. 2005.

[11] J. Zhu, R. Liu, Z. Jiang, P. Wang, Y. Yao, and Z. Shen. Optimization of drug regimen in chemotherapy based on semi-mechanistic model for myelosuppression. *Journal of biomedical informatics*, 57:20–27, Oct. 2015.
